# Mr. Cooper, on the Radical Cure of Bubonocele

**Published:** 1803-04-01

**Authors:** Samuel Cooper

**Affiliations:** Upper Charlotte Street, Fitzroy Square


### *To the Editors of the Medical and Phyſical Journal.*

GENTLEMEN,

YOUR request to know the name of the Author of the "Considerations to shew that the idea of effecting a radical cure of the Bubonocele ought not to be entirely abandoned," inserted in the 48th Number of your Journal, turns immediately into a sort of compulsion, when you add that otherwise you shall be under the necessity of giving the priority priority of a proposal in them to Mr. Carlisle, who is stated publicly to have signified his intention to perform such an operation at the weekly consultation of the Westminster Hospital, seven weeks before the receipt of S. C's. letter. Placed in this situation, it cannot for a moment be a matter of hesitation with me to declare, that I am the Author of the above paper:—it is done the more willingly, because, at the same time, I shall take the opportunity to shew that my communication is totally independent of Mr. Carlisle, and to explain, not only that this gentleman posesses no right to the priority of the proposal superior to mine, but also that I do not mean to claim it myself as an original innovation in surgery.

It was more than two years ago, during my apprenticeship to Mr. Ramsden, of St. Bartholomew's Hospital, that the idea of the possibility of radically curing the Bubonocele first struck me, and which, in the course of conversation, I more than once mentioned to other surgical students: But my thoughts upon this subject had become quite extinct till about five months ago, when finding, on the perusal of Mr. Sharp's Critical Enquiry, that this excellent surgeon had not rejected the practicability of radically curing the Bubonocele, my former considerations naturally revived; and, as early as this, I communicated my opinion to some others, and more particularly to Mr. Lawrence, who demonstrates anatomy at the above hospital. At length I wrote the little paper contained in your Journal, rather with a view to excite others to employ their superior talents in a further investigation of the matter, than on account of any value placed upon that trifling production. In extending our researches back into former writers upon surgery, we shall discover that no small share of those inventions, claimed by surgeons of the present day, are actually not modern ; and either that some strong allusions, leading to them, have been thrown out in the works of their predecessors, or, what is more, that an open description of them is to be met with. Whatever vanity might once have been entertained on my part, concerning the origin of the proposal, certain it is that it was soon dispelled by the more philosophical pleasure which I derived from learning that a similar operation had both been antecedently proposed, and often practised with great success. For this information we need only refer to the second volume of Heister's Surgery, p. 110. Nor must it be forgotten here to notice the opinion of Le Dran, who thought that a ligature might be made on the hernial sac in in the hernia cruralis and inguinalis in women, but not for the latter case in men, for fear of injuring the testicle. See the remarks following his 58th Observation. The names of several other writers might be cited to prove, either that the possibility of radically curing hernia by the proposal in question had been conceived by them, or successfully practised. Let it be sufficient just to observe, that Mr. Latta, in his System of Surgery, describes a like mode of radically curing the Bubonocele, (Vid. Vol. I. p. 285) who, though he does not venture to recommend it on his own experience, says, that it is founded in reason, and is certainly well worth the attention of surgeons.

Saviand's method of procuring a permanent cure of the exomphalos, for the antiquity of which we must mount up to Celsus, in point of principle, bears so great similitude to this operation, that we ought not to look upon it as unconnected with the present subject; and if it be true, that he might have operated more elegantly by first dividing the integuments, and then applying his ligature round the hernial sac, the very fact of its being done in not the most advantageous style, and yet successfully, tends to prove in a more convincing manner, that a ligature may be applied, at least on young subjects, in this way, without a bad event. (See his Nouveau Recueil d'Observations Chirurgicales.)

Desault also cured nine cases of umbilical herniæ in children by that mode, and Celsus advised it to be attempted only from the age of seven to fifteen; it is true, however, as Sabbatier observes, De la Medicine Operatoire, tom. i. p. 128, it may be doubted whether they continued permanently cured.

It only remains for me to express how happy I am in having done this justice to the above authors, which, had I known their claims, would have been rendered them before; it is no less gratifying to me that Mr. Carlisle has announced his intention to do an operation which appears never to have been properly* put to the test of experience in this country, and which reason and the interests of society, and the little we have yet on record concerning it, seem to demand.

*Upper Charlotte Street, Fitzroy Square.* I am, &c.

*March* 7, 1803. SAMUEL COOPER.

---

* See our Address to Correspondents at the end of this Number. Ed.

Case

